# Addressing the legal and health challenges of licensed medical cannabis users who want to travel abroad

**DOI:** 10.1186/s13584-025-00723-2

**Published:** 2025-10-27

**Authors:** Hagit Bonny-Noach, Vered Ne’eman-Haviv

**Affiliations:** 1 Department of Criminology, Faculty of Social Sciences and Humanities, Ariel University, Ariel, Israel; 2 The Israeli Society of Addiction Medicine, Ramat Gan, Israel

**Keywords:** Medical cannabis, Travel, Regulations, Health risk, Legality

## Abstract

**Background:**

Despite the global rise in medical cannabis use for health purposes, patients with medical cannabis licenses face significant legal and health risks when traveling abroad. The present study sought to understand how medical cannabis users experience these challenges while traveling abroad.

**Methods:**

We conducted semi-structured interviews with 15 Israeli medical cannabis license holders and collected supplementary data from two of the largest Facebook groups of medical cannabis users in Israel.

**Results:**

Two main themes were identified : (a) **vague regulations for medical cannabis users who travel abroad.** Patients described international regulations as vague, with authorities failing to treat medical cannabis like other prescription medications for tourists. They criticized the lack of clear, formal information and guidelines; (b) **legal**,** semi-legal**,** and illegal solutions adopted by medical cannabis users traveling abroad.** In response to these challenges, patients have adopted various strategies, such as avoiding travel, selecting cannabis-friendly destinations, planning in advance to obtain cannabis through legal or illegal means, and finding alternative self-medication options.

**Conclusion and policy recommendation:**

The study revealed the need for improving the clarity of international policies to reduce legal and health risks faced by licensed medical cannabis users traveling abroad. We recommend that countries adopt mutual recognition of prescriptions, standardized travel certificates, and improved institutional guidance. The World Health Organization (WHO) should support this effort by issuing non-binding global guidelines and promoting best practices. Grounding these recommendations in access to care and health equity frameworks would ensure fair, safe, and continuous treatment access across borders.

## Introduction

Medical cannabis use for health purposes is increasing worldwide, with growing recognition of its therapeutic benefits. Patients turn to medical cannabis for various conditions, including severe or chronic pain, epilepsy, insomnia, PTSD, and to improve health-related quality of life [1–3]. As of 2020, 64 countries had established provisions in their national legislation or developed guidelines permitting the medical use of cannabinoid pharmaceutical preparations or cannabis-based products to treat a variety of medical conditions [4]. The increase in the number of countries regulating medical cannabis reflects a global trend toward the acceptance of cannabis for therapeutic purposes. Canada, Germany, the Netherlands, and most of the states in the US have well-established medical cannabis regulations [5], but in many countries, cannabis remains illegal, including for medical purposes.

Medical cannabis laws vary across countries. Since the 1990 s, Israel has become one of the leaders in the medical cannabis industry [6, 7]. Over the years, the number of Israeli patients with medical cannabis prescriptions has steadily risen, reaching approximately 120,000 licensed patients by 2024 [8]. However, despite being licensed to possess medical cannabis, these patients experience unique challenges when they come to fly abroad. Although traveling abroad is a deeply ingrained and frequent part of Israeli life, and approximately 9 million departures abroad of Israelis were recorded in 2023 (in a population estimated at 9.915 million people) [9], no formal Israeli guidelines currently exist for medical cannabis patients traveling abroad.

The challenges faced by Israeli medical cannabis patients reflect broader international issues. For example, medical cannabis patients in the Schengen Area face legal uncertainty when traveling, despite mechanisms for controlled substances. Under Article 75 of the Schengen Convention, travelers may carry narcotic medications across Schengen borders using a special certificate valid for up to 30 days [10]. Yet, not all Schengen countries recognize medical cannabis under this framework. Some countries, for example, the Netherlands and Germany, allow medical cannabis under regulated conditions, but others, including France and Sweden, either restrict or do not fully recognize its medical use. Furthermore, in Canada, although domestic use by adults is permitted, exporting or importing medical cannabis remains illegal, posing significant challenges for patients who travel [11]. These examples demonstrate the global inconsistencies in medical cannabis regulations and attest to the need for international dialogue and policy development to support patient mobility. To our knowledge, comprehensive international data on medical cannabis patients traveling abroad and related border incidents is limited. Countries do not systematically track or publish cases of travelers facing legal issues because of carrying prescribed medical cannabis, making it difficult to gauge the true scale of the problem.

While some research has concentrated on recreational cannabis use among travelers [12–14], the unique challenges faced by medical cannabis patients when navigating varying international laws remain underexplored. To our knowledge, this is the first study to specifically address the use of medical cannabis by travelers. Given the limited understanding of the legal and health challenges medical cannabis patients face when traveling abroad, this study aims to explore these challenges from the users’ perspective. By giving voice to the lived experiences of licensed medical cannabis patients, this study provides insight into how patients manage uncertainty, legal precarity, and the need for continuity of care when crossing borders. These insights can inform future policy discussions, improve patient guidance, and promote greater awareness among health professionals, regulators, and travelers themselves.

## Methods

The topic of international travel by medical cannabis patients emerged in the course of a broader qualitative study on cannabis diversion by Israeli medical cannabis patients. Although traveling abroad was not an initial focus of the original study, the issue came up already in the first interview as a key concern and source of difficulty for Israeli medical cannabis patients. Recognizing the significance of the issue and given the first author’s research background in tourism and substance use, the interview guide was expanded to include targeted questions about international travel. This allowed for an in-depth exploration of what became a central and recurring issue to understand in the interviews. We conducted semi-structured interviews and asked participants about their perceptions, behaviors, and difficulties related to traveling abroad as medical cannabis patients. Participants were selected using purposive sampling [15]. We interviewed 15 participants, ten of them women, who reported holding valid Israeli medical cannabis licenses, aged 25–60 years. Participants were recruited through online posts on cannabis patient forums and Facebook groups, by word of mouth, and snowball sampling. The main inclusion criteria were being over 18 years old and holding a valid license for medical cannabis. Because traveling abroad is a frequent part of Israeli life [9], most of the participants had traveled abroad or intended to do so while holding a medical cannabis license. The researchers alternated in conducting the interviews, following a semi-structured interview guide to ensure consistency and methodological sensitivity throughout the process. Interviews were conducted by Zoom (Zoom Video Communications Inc., San Jose, CA), which was linked to the secured cloud system of the university to ensure encrypted, safe, and institutionally approved communication. Each interview lasted approximately 60–90 min. All interviews were audio-recorded with the participants’ consent and transcribed verbatim for analysis. We determined that we reached data saturation when additional interviews no longer yielded new themes or insights. We used thematic analysis to interpret the data [16], and conducted the coding process manually. First, we read all transcripts in their entirety to gain a general understanding of the content. Next, we generated initial codes to capture key ideas and experiences expressed by the participants. We then grouped the codes into broader themes that reflected the main challenges and experiences shared by the participants. Themes were actively constructed through interpretive engagement with the data rather than passively emerging from it [17, 18]. Our analysis was informed by the researchers’ disciplinary backgrounds in criminology, drug policy, tourism, and substance use. To ensure reliability, the two researchers coded the transcripts independently and then compared and discussed their coding to reach a consensus on the emerging themes.

We also collected supplementary data from two of the largest Facebook groups for medical cannabis users in Israel. These public groups were selected based on membership size and level of activity. We used keyword searches (e.g., “Travel abroad,” “Flight”) to identify relevant posts and discussions from the past three years. Publicly available posts were thematically analyzed to triangulate findings with interview data. No personal identifiers or private messages were accessed or used.

The study was conducted in accordance with the ethical guidelines of the authors’ institution, and ethical approval was obtained from the institutional review board (IRB) (AU-SOC-VNH − 20220622). Informed consent was obtained from all participants before their involvement in the study. To ensure anonymity, identifying information was removed from the transcripts, and participants were assigned pseudonyms.

## Results

Two main themes were identified from the interviews with medical cannabis users scheduled to travel abroad:

### First theme: vague regulations for medical cannabis users who travel abroad

This theme reflects the challenges faced by medical cannabis users when traveling abroad. Participants described international regulations as vague and burdensome, with authorities failing to treat medical cannabis like other prescription medications for tourists. Unlike other controlled medications (e.g., painkillers, insulin), medical cannabis is often treated by authorities inconsistently and is subject to diverse legal interpretations, which can create challenges for tourists.

Shiri described the difficulty that she faced traveling with her cannabis and the differences between her as a medical cannabis patient and other patients carrying different types of medicines:

A person, in general, who’s dealing with, say, some other disease [and another medicine] and wants to fly abroad, just orders a ticket and flies; all he needs is money and a hotel and a flight.

Participants also reported that the process of obtaining approval for an Israeli medical cannabis license abroad was overly complex, even in countries that have mechanisms to allow patients to bring in prescribed medical cannabis with notarized documents. Participants criticized the lack of clear formal information and guidelines from global and local authorities for patients traveling with medical cannabis. Tourists who use medical cannabis need to cope with legal and administrative hurdles to obtain temporary access to cannabis in the destination country, even if they travel for short durations. In both Facebook groups, users of medical cannabis often asked questions to clarify how to behave when flying abroad. For example, Sigal asked: “Can I take a small bottle of [cannabis] oil on the flight?” She received various answers because the information available appeared to be confusing and vague. One answer suggested: “Don’t listen to anybody’s recommendation if you don’t want to end up in jail.”

Dori, another user, asked:

Has anyone here managed to fly with medical cannabis and can help with the bureaucracy? On the websites I read, there are all kinds of instructions about certain countries, like in the case of the Czech Republic, for example, you need to talk to the embassy, but in practice, when I call them, they have no idea what I’m talking about. They just transferred me from one to the other.

Eran responded to Dori’s question, explaining that when he traveled to Germany earlier the same year, the embassy failed to provide clear guidance. He had to reach out directly to the German Ministry of Narcotics, but their instructions were also ambiguous.

Some of the interviewees raised the concern that the medical cannabis licenses of Israeli patients might not be recognized when they travel abroad. This creates legal grey areas for Israeli patients, who must learn to comply with the requirements of foreign legal systems or do without medical cannabis during international trips. Liron said:

As far as I know, in most countries, it’s not possible [to receive medical cannabis]. The prescription is for Israel and that’s it.

The questions medical cannabis users raised on Facebook and those mentioned by study participants attest to the ambiguity and lack of clarity surrounding the laws and regulations regarding what is allowed or prohibited when trying to use a medical cannabis license abroad. Many countries lack clear guidelines or accessible information about how tourists should handle medical cannabis when entering or staying there. The lack of transparency leaves patients uncertain about whether they can bring their cannabis, what documentation they need, and how to avoid negative legal consequences. Some may end up making dangerous decisions, such as smuggling cannabis or going without it.

### Second theme: legal, semi-legal, and illegal solutions adopted by medical cannabis users traveling abroad

Because of the legal and health challenges identified in the previous theme, medical cannabis users have developed various strategies to cope with the complexities of traveling abroad and their need for cannabis. Some indicated that because of the problematic situation, they avoided traveling abroad. Orna said:

I can tell you that I am on the endometriosis Facebook group and we have had this conversation about how to go on vacation [abroad] many times, and many of us avoid going on vacation at all… and if they go, then they prefer vacation in Israel… Like what, they’ll catch you smoking at the Dead Sea and what will they do to you?

Others mentioned that they selected cannabis-friendly country destinations and mostly avoided traveling to countries where cannabis was forbidden. Kevin said:

If I hear about a trip and I know there will be a problem, I’ll take that into account and I might not go to that destination… If I hear that it’s forbidden, I won’t go there despite everything. I’m not one of those people.

Almost all interviewees mentioned Amsterdam as a cannabis-friendly destination that they preferred to visit when traveling abroad. Orna said she opted to travel to Amsterdam even if it cost more than other countries and even if it was colder than other places:

For my birthday, I’m flying to Amsterdam… a place where I can use cannabis… It’s more expensive in Amsterdam… this story isn’t cheap at all… I hope that when I come to Amsterdam, I’ll be more relaxed and it will be easier for me and I’ll be able to take walks and deal with this thing and have fun and that’s it. Like, I’m a little afraid of the cold and all this and what it will do to my body because I have such a rheumatic disease, so there are these concerns.

Participants described planning in advance how to access cannabis at their destination, either by selecting a cannabis-friendly destination like Amsterdam or by arranging to obtain cannabis through legal or illegal means upon arrival. The legal planning in advance to obtain cannabis included researching to understand the cannabis laws in the destination country, seeking permits or approvals, and finding out whether they could travel with their own cannabis. Even when medical cannabis programs are legal in the destination country, there may be complex requirements for obtaining permission for medical cannabis during travel. Patients may have to deal with customs and border control, which often have strict regulations about bringing cannabis into the country, even with medical documentation. Shiri described how she had to plan and get organized to handle the bureaucracy involved:

In order to fly, I have to plan well ahead of time, I have to send consular requests to bring cannabis into that country, and I need the country to be prepared to accept Israeli medical licenses. Let’s check how to do it, wait, don’t do it too quickly, let’s check which country you can go to with your license, and let’s check how to do it. It takes me longer.

Some described advance planning of how to obtain cannabis upon arrival in to the destination country, both semi-legal or illegal. Alona said:

I try to prepare in advance and arrange to have it [the cannabis] there when I land. Whether it’s someone I know or someone else, I make sure to have the cannabis waiting for me on arrival. I try to prepare ahead of time.

When legal access to cannabis is not possible, some patients might research black market sources or networks for cannabis in the destination country and plan to act illegally. Iris described how other medical cannabis patients bought cannabis illegally in the country of destination: “They buy it on Telegras [an Israeli cannabis marketplace on Telegram]… they take a risk…” Noel indicated:

I managed there, I just inquired before and I managed… Today it’s everywhere… I talked to a friend who knows best about places abroad and I told him, listen, I’m flying to Vienna, what am I doing? So he told me, go to some bar… don’t go to strangers…”.

Others indicated that their solution was to find alternative self-medication options. Nofar said that when traveling abroad, she took other drugs for the trip rather than cannabis:

But there are solutions, look, I didn’t want to take these pills on a regular basis because I know they are very harmful to the body, but if it’s once in a while for a certain period like this [traveling abroad]….

Iris noted that she traveled with prescription opioid morphine syrup as a substitute for cannabis:

Three weeks ago, I flew with my mother and my sisters to Bucharest for six days. I was without cannabis and was perfectly fine. I compensated myself with morphine syrup… I got really high on morphine during the days I was in Bucharest, but I didn’t feel any urge for cannabis.

When the interviewer asked her whether prescription morphine was not against the law, she answered that, to her knowledge, there were no problems with it abroad. When the interviewer suggested that prescription opioids were also not allowed in all countries, she said:

I’ve been flying with morphine for years… [I’ve been to] Thailand and all over Europe… I’m hearing this for the first time.

Noel described how her substitutes for cannabis included mental preparation and meditation as self-medication:

“If now I fly to a place without cannabis, I first prepare myself mentally… to be ready for it, I do meditation… I have a lot of ways to deal with anxiety, let’s say”.

Others mentioned alcohol and cigarettes as substitutes. Merav said that if she did not have cannabis abroad, she drank alcohol instead:

I’ll drink some wine… Drink beer, drink wine, something that will bring down the anxiety level.

Merav and other participants described alcohol as a temporary substitute for medical cannabis for use abroad in coping with anxiety. This illustrates the blurred boundaries between medicinal and affective use of medical cannabis. Although medical cannabis is not officially indicated for treating anxiety in Israel, participants framed it as part of their personal emotional regulation toolkit. In this sense, alcohol functioned less as a purely recreational choice and more as an improvised coping mechanism and self-medication. The mention of cigarettes as a substitute for medical cannabis by some of the participants may reflect underlying nicotine dependence or a familiar self-soothing behavior, such as smoking, rather than a direct medical replacement for cannabis.

## Discussion

The present study sought to understand the legal and health challenges that Israeli licensed medical cannabis users faced while traveling abroad. The findings attest to the complex challenges these patients encountered, revealing significant gaps in international regulatory frameworks and the support available to travelers. This discussion situates the findings within broader debates on global health governance, access to care, and the right to treatment continuity across borders. The first theme identified in the interviews reveals the inconsistency and complexity of international regulations governing the use of medical cannabis. Participants frequently described these regulations as unclear and burdensome, lacking standardized processes for recognizing medical cannabis licenses across borders. This created confusion and stress for medical cannabis users, who had to cope with various legal systems without sufficient guidance from either their home country or destination authorities. This pattern is not unique to Israeli travelers and illustrates a broader structural gap faced by medical cannabis users worldwide when they cross jurisdictions with divergent laws.

The second theme revealed in the analysis of the interviews is concerned with the various strategies adopted by medical cannabis users in response to these legal and health challenges. The study found that some participants avoided traveling abroad or chose cannabis-friendly destinations, seeking legal or semi-legal means of gaining access to cannabis abroad, and even turning to illegal methods. The use of similar semi-legal or illegal methods and strategies has been reported in Israel, where some participants mentioned obtaining cannabis through informal networks such as the Telegras platform on Telegram.

This regulatory patchwork forces patients to devise ad hoc solutions, often at the expense of safety and continuity of care. This finding is consistent with those of previous studies, which have shown that the absence of clear and consistent medical cannabis policies across international borders, not limited to tourism, leads to conflicts with the law and disrupts the continuous medical care patients require [2, 19].

The findings of this study can be understood through the theoretical lenses of access to care [20] and health equity [21, 22], both of which are central to health policy and global public health discourse but apply primarily to conditions within national health systems and do not address cross-border care. According to AHRQ, access to care is not limited to the physical availability of services but includes also the ability to seek, reach, and successfully use healthcare services to meet medical needs [20]. The challenges faced by licensed medical cannabis users who travel abroad range from unclear regulations to the risk of infringing on these dimensions of access. These barriers affect a vulnerable patient group that relies on the continuous supply of medical cannabis for chronic conditions such as PTSD, cancer-related pain, and neurological disorders. Similarly, the WHO defines health equity as “the absence of unfair and avoidable or remediable differences in health among population groups” [22]. Yet the inconsistent international treatment of medical cannabis, especially when contrasted with other controlled prescription drugs such as opioids, reveals a regulatory gap that disrupts the continuity of care and undermines equity for patients crossing national borders. The WHO or similar international bodies could collect and disseminate accurate, up-to-date information on each member state’s medical and recreational cannabis regulations. Making such information publicly available would enable patients, healthcare providers, and policymakers to plan more effectively for cross-border care without expecting universal legal harmonization.

Health authorities in countries like Israel, where medical cannabis policies are already in place, could assume greater responsibility for collecting and disseminating information about cannabis regulations in destination countries. For example, the Israeli Ministry of Health could maintain a publicly accessible online database summarizing the cannabis policies of the main travel destinations. Additionally, as part of a broader harm reduction approach, these services should inform patients about the risks of illicit cannabis use and of resorting to opioids as substitutes. International carriers could also integrate this information into pre-travel guidance materials, similarly to how they provide information on customs restrictions for other controlled medications.

International travelers are generally expected to familiarize themselves with the legal requirements of their destination, including customs regulations, visa policies, and restrictions on controlled substances. This expectation also applies to medical cannabis. Unlike other prescribed medications, the legitimate use of medical cannabis is frequently not recognized in certain jurisdictions. Whether travelers should self-declare their use or possession of medicinal cannabis at airport security, sniffer dog inspections, and customs stations remains a legally and ethically complex issue. Disclosure may reduce suspicion but also expose travelers to legal consequences, depending on the jurisdiction.

Another issue of concern for licensed medical cannabis users who travel internationally is driving. To our knowledge, no country explicitly permits driving under the influence of cannabis, even when medically prescribed. This presents an additional legal risk for medical cannabis users who need to drive while traveling abroad.

Although this study focused on Israeli medical cannabis users, the policy recommendations presented here are relevant to all international travelers who rely on medical cannabis.

**Limitations and future directions**: The present study does not include a comprehensive analysis of the institutional and regulatory landscape governing the use of medical cannabis. State institutions such as ministries of health, customs authorities, and drug regulatory agencies play a critical role in shaping patients’ cross-border access to medical cannabis through the enforcement of national and international drug control conventions. The study is further limited by its small, purposive sample that is not representative of licensed medical cannabis users in Israel, which restricts the generalizability of the findings. It relies on self-reported data that may be affected by recall bias or social desirability, and it reflects the Israeli context, which may differ markedly from other countries. Future research should explore the legal and institutional systems concerning medical cannabis use across borders to strengthen patient-centered findings and support the development of clear and consistent global health policies.

**In conclusion**, this study explored the increasingly significant challenge faced by medical cannabis patients who travel internationally: the lack of consistent regulations across borders, which exposes them to legal risks and disrupts continuity of care. The findings are relevant to all individuals crossing national borders who depend on medical cannabis. Our findings reveal the need for practical, coordinated solutions, such as improved information-sharing by national ministries of health, standardized documentation protocols, bilateral or regional agreements between countries with medical cannabis programs, and global information platforms led by organizations like the WHO, to reduce uncertainty and protect patients. Strengthening these measures can provide immediate benefits for travelers, bridging gaps in access without requiring full harmonization of laws. Although future research is needed to further explore institutional and cross-national frameworks, the evidence presented here demonstrates that several realistic policy steps can already be implemented to improve patient safety, continuity of care, and overall quality of life for medical cannabis travelers worldwide.

### Policy recommendations

Given the fragmented and inconsistent international legal landscape concerning medical cannabis, we recommend a series of pragmatic and coordinated measures designed to improve continuity of care and reduce legal risks for patients who travel across borders: (a) negotiating bilateral or regional agreements that allow the controlled mutual recognition of medical cannabis prescriptions, similar to existing frameworks for other controlled substances; (b) creating standardized travel certificates for patients, endorsed by their national ministries of health, to provide border officials with a clear and verifiable document of legitimate medical use; (c) developing publicly accessible online portals managed by ministries of health to provide up-to-date information about cannabis regulations in common travel destinations; international health organizations, particularly the WHO, could serve as global information hubs by collecting and disseminating member states’ cannabis regulations and best practices to reduce uncertainty for patients and clinicians; (d) the same information should also be disseminated to patients through healthcare providers, pharmacies, and international carriers; (e) integrating harm-reduction messaging into all patient-facing guidance, including clear warnings about the risks of illicit purchase abroad, substitution with opioids or other substances, and driving under the influence of cannabis even when prescribed. These coordinated efforts would benefit medical cannabis users worldwide who travel internationally.

Innovative practical solutions could further support medical cannabis patients who travel abroad. These include (a) developing a multilingual mobile application, sponsored by national health authorities, to provide real-time updates on cannabis regulations in destination countries and guidance on required documentation; (b) introducing a secure digital patient certificate containing verified prescription details and dosage information to be presented at borders or pharmacies; (c) creating dedicated travel-insurance products tailored to the needs of medical cannabis patients to cover potential legal or medical complications related to carrying their medication; and (d) implementing joint information campaigns in collaboration with airports and airlines to ensure that patients receive clear instructions on legal requirements and safe transport of their prescribed medication. Such innovations, alongside coordinated policies, can help reduce uncertainty, improve continuity of care, and enhance the safety and wellbeing of patients who rely on medical cannabis while traveling internationally.

## Data Availability

The database for this study is not publicly available. It can be obtained from the authors on reasonable request.
